# Commentary: Editorial: Significant influencing factors and effective interventions of mobile phone addiction

**DOI:** 10.3389/fpsyg.2022.957163

**Published:** 2022-09-16

**Authors:** Xavier Carbonell, Tayana Panova, Arnau Carmona

**Affiliations:** FPCCE Blanquerna, Universitat Ramon Llull, Barcelona, Spain

**Keywords:** smartphone, smartphone addiction, treatment, mobile phone (or smartphone) use, psychological disorder

In their editorial introducing the research topic *Significant Influencing Factors and Effective Interventions of Mobile Phone Addiction*, Liu et al. (2022) declare that no intervention research has been published on the research topic about smartphone addiction, but they suggest that intervention on smartphone addiction is still a key research focus. In fact, since smartphones have changed the communication and information landscape, social and scientific concern about smartphone addiction has been ongoing (Bianchi and Phillips, 2005; Chóliz, 2010) and hundreds of papers have been published about smartphone addiction (Carbonell et al., 2009, 2016; Olson et al., 2022). However, there is also significant criticism about the nature of this concept. Many of the studies about “smartphone addiction” use correlational methodology with several limitations such as: (a) a lack of longitudinal studies; (b) screening instruments that are not valid for diagnosis; (c) a large probability of false positives; (d) exploratory studies relying on self-report data; (e) the use of convenience samples of university and secondary students; (f) a lack of clinical samples; and (f) a lack of consistency in methodology, definitions, measurement, and diagnostic criteria across studies [see the critical reviews of (Pedrero et al., 2012; Billieux et al., 2015a,b; Panova and Carbonell, 2018)]. In short, the principal limitation is that these studies confuse addiction-like symptoms with a real disorder. By doing so, we risk over-pathologizing daily life (Billieux et al., 2015b) and undermining the seriousness of psychiatric disorders (Petry and O'Brien, 2013).

With the smartphone, *via* the Internet, it is possible to access a variety of apps and websites such as social media, pornography, video games and gambling, some of which contribute to increased feelings of dependence, suggesting that rather than being addictive *per se*, smartphones are the medium that enable engagement with potentially addictive activities (Kuss et al., 2018; Lowe-Calverley and Pontes, 2020) and are associated with poor health outcomes (Chen et al., 2020). Problematic smartphone use can be viewed as generalized (see Brand et al., 2014) or predominantly specific. From this specific position, Lopez-Fernandez et al. (2017) points out that the term “mobile phone dependence” could be an inadequate construct because people are not dependent on the mobile phone but on the activities that can be performed with it. Moretta et al. (2022) arrives at a similar conclusion when she argues that an appropriate approach would involve focusing on the behavior and not on the device (smartphone). One way to overcome this taxonomical controversy is to split the internet use disorder into predominantly mobile and predominantly non mobile (Montag et al., 2021). If we compare this to the well-known field of substance addiction, it would be analogous to confusing addiction to the bottle with addiction to the alcohol (Kuss and Griffiths, 2017), or a fixation on the needle (Miller, 2005) rather than the drug itself (Panova and Carbonell, 2018), or with the obsolete terminology of “computer addiction” (Shotton, 1989). In the words of Lowe-Calverley and Pontes (2020) “smartphone users are likely to become addicted to the functionalities they access on their smartphones (*content*) and not the smartphones themselves (*medium*)”. Furthermore, there is a strong overlap between self-perceived smartphone addiction and Internet and social media use (Carbonell et al., 2018; Chen et al., 2020). A recent narrative review suggests that problematic use of the smartphone could involve various forms of problematic use of the internet such as gaming, gambling, social media, etc. (Fineberg et al., 2022).

Obviously, the smartphone plays an important role in allowing for the easy, convenient, private and fast access to the Internet, thus increasing the addictive potential of some behaviors because they are more available and accessible. In some ways, smartphones and cigarettes are analogous. Smoking tobacco is believed to have begun as early as 5000–3000 BC in South America and was introduced to Europe in the late 16th century. However, the use of cigarettes and nicotine addiction climbed markedly when James Albert Bonsack developed a cigarette-making machine in the 1880's which vastly increased the production of cigarettes (Wikipedia, 2022). Cigarettes were one of the most commonly traded industrial products in the 20th century and their price, availability and social acceptance popularized the use of nicotine. However, as is well-known, the problem is not the cigarette itself: the addictive substance is the nicotine. Again, the confusion between the medium and the content suggest that the problem is the behavior (e.g., gambling), not the smartphone itself.

To return to the original point of our commentary – a recent study insists on one critical point: the absence of clinical samples. Pedrero-Pérez et al. (2022) carried out a systematic review of articles that have applied some type of treatment for smartphone addiction. To their surprise, up to September of 2020 there had only been two articles published that met the criteria. Both articles provided minimal scientific evidence and the review concluded that “Despite the fact that mobile phone addiction has been discussed for 15 years, and at times with apocalyptic overtones, not a single reliable study has been found that offers a therapeutic response which can be empirically contrasted.”

The lack of evidence is reinforced when we study the psychometric validity of smartphone addiction scales. They conclude that many technology measures appear to measure a similar, poorly defined construct that sometimes overlaps with pre-existing measures of wellbeing” (Shaw et al., 2020; Davidson et al., 2022) and that smartphone addiction scales were generally good at identifying who believes themselves to be addicted, although they do not reflect objective smartphone use (Geyer et al., 2021).

To summarize, the absence of people asking for treatment, the confusion of the medium with the content, the lack of psychometric validity and the absence of objective measures strongly suggest that, after 15 years of research, 2022 may be the year that marks the end of the smartphone addiction debate, encouraging us to refocus clinical and research efforts on real disorders.

## Author contributions

XC initiated and drafted the general commentary. TP and AC contributed theoretical background and feedback. All authors approved the final version of the manuscript for submission.

## Conflict of interest

The authors declare that the research was conducted in the absence of any commercial or financial relationships that could be construed as a potential conflict of interest.

## Publisher's note

All claims expressed in this article are solely those of the authors and do not necessarily represent those of their affiliated organizations, or those of the publisher, the editors and the reviewers. Any product that may be evaluated in this article, or claim that may be made by its manufacturer, is not guaranteed or endorsed by the publisher.

## References

[B1] Bianchi A. Phillips J. G. (2005). Psychological predictors of problem mobile phone use. Cyberpsychol. Behav. 8, 39–51. 10.1089/cpb.2005.8.39 15738692

[B2] Billieux J. Maurage P. Lopez-Fernandez O. Kuss D. J. Griffiths M. D. (2015a). Can disordered mobile phone use be considered a behavioral addiction? An update on current evidence and a comprehensive model for future research. Curr. Addict. Rep. 2, 156–162. 10.1007/s40429-015-0054-y

[B3] Billieux J. Schimmenti A. Khazaal Y. Maurage P. Heeren A. (2015b). Are we overpathologizing everyday life? A tenable blueprint for behavioral addiction research. J. Behav. Addict. 4, 119–123. 10.1556/2006.4.2015.009 26014667 PMC4627665

[B4] Brand M. Young K. S. Laier C. (2014). Prefrontal control and internet addiction: a theoretical model and review of neuropsychological and neuroimaging findings. Front. Hum. Neurosci. 8, 1–13. 10.3389/fnhum.2014.00375 24904393 PMC4034340

[B5] Carbonell X. Chamarro A. Oberst U. Rodrigo B. Prades M. (2018). Problematic use of the Internet and smartphones in university students : 2006 – 2017. Int. J. Environ. Res. Public Health 15, 1–13. 10.3390/ijerph15030475 29518050 PMC5877020

[B6] Carbonell X. Guardiola E. Beranuy M. Bellés A. (2009). A bibliometric analysis of the scientific literature on Internet, video games, and cell phone addiction. J. Med. Libr. Assoc. 97, 102–107. 10.3163/1536-5050.97.2.006 19404500 PMC2670219

[B7] Carbonell X. Guardiola E. Fuster H. Gil F. Panova T. (2016). Trends in scientific literature on addiction to the internet, video games, and cell phones from 2006 to 2010. Int. J. Prev. Med. 7, 63. 10.4103/2008-7802.179511 27141282 PMC4837796

[B8] Chen I. H. Pakpour A. H. Leung H. Potenza M. N. Su J. A. Lin C. Y.. (2020). Comparing generalized and specific problematic smartphone/internet use: Longitudinal relationships between smartphone application-based addiction and social media addiction and psychological distress. J. Behav. Addict. 9, 410–419. 10.1556/2006.2020.00023 32592655 PMC8939406

[B9] Chóliz M. (2010). Mobile phone addiction: a point of issue. Addiction 105, 373–374. 10.1111/j.1360-0443.2009.02854.x 20078493

[B10] Davidson B. I. Shaw H. Ellis D. A. (2022). Fuzzy constructs in assessment: The overlap between mental health and technology “use.” *Open Sci. Framework* 133, 107206. 10.1016/j.chb.2022.107206

[B11] Fineberg N. A. Menchón J. M. Hall N. Dell'Osso B. Brand M. Potenza M. N.. (2022). Advances in problematic usage of the internet research – a narrative review by experts from the European network for problematic usage of the internet. Compr. Psychiatry 118, 152346. 10.1016/j.comppsych.2022.152346 36029549

[B12] Geyer K. Carbonell X. Beranuy M. Calvo F. (2021). Absence of objective differences between self-identified addicted and healthy smartphone users? Int. J. Environ. Res. Public Health 18, 3702. 10.3390/ijerph18073702 33916256 PMC8037484

[B13] Kuss D. Harkin L. Kanjo E. Billieux J. (2018). Problematic smartphone use: Investigating contemporary experiences using a convergent design. Int. J. Environ. Res. Public Health 15, 142. 10.3390/ijerph15010142 29337883 PMC5800241

[B14] Kuss D. J. Griffiths M. D. (2017). Social Networking Sites and addiction: ten lessons learned. Int. J. Environ. Res. Public Health. 14, 311. 10.3390/ijerph14030311 28304359 PMC5369147

[B15] Liu Q. Zhou Z. Eichenberg C. (2022). Editorial: Significant influencing factors and effective interventions of mobile phone addiction. Front. Psychol. 13, 909444. 10.3389/fpsyg.2022.909444 35572302 PMC9096215

[B16] Lopez-Fernandez O. Kuss D. Romo L. Morvan Y. Kern L. Graziani P.. (2017). Self-reported dependence on mobile phones in young adults: a European cross-cultural empirical survey. J. Behav. Addict. 6, 168–177. 10.1556/2006.6.2017.020 28425777 PMC5520117

[B17] Lowe-Calverley E. Pontes H. M. (2020). Challenging the concept of smartphone addiction: An empirical pilot study of smartphone usage patterns and psychological well-being. Cyberpsychol. Behav. Soc. Netw. 23, 550–556. 10.1089/cyber.2019.0719 32498607

[B18] Miller J. (2005). Heroin addiction: the needle as transitional object. J. Am. Acad. Psychoanal. 30, 293–304. 10.1521/jaap.30.2.293.21955 12197257

[B19] Montag C. Wegmann E. Sariyska R. Demetrovics Z. Brand M. (2021). How to overcome taxonomical problems in the study of Internet use disorders and what to do with “smartphone addiction”? J. Behav. Addict. 9, 908–914. 10.1556/2006.8.2019.59 31668089 PMC8969715

[B20] Moretta T. Buodo G. Demetrovics Z. Potenza M. N. (2022). Tracing 20 years of research on problematic use of the internet and social media: Theoretical models, assessment tools, and an agenda for future work. Compr. Psychiatry 112, 152286. 10.1016/j.comppsych.2021.152286 34749058

[B21] Olson J. A. Sandra D. A. Colucci É. S. al Bikaii A. Chmoulevitch D. Nahas J.. (2022). Smartphone addiction is increasing across the world: a meta-analysis of 24 countries. Comput. Hum. Behav. 129, 107138. 10.1016/j.chb.2021.107138

[B22] Panova T. Carbonell X. (2018). Is smartphone addiction really an addiction? J. Behav. Addict. 7, 252–259. 10.1556/2006.7.2018.49 29895183 PMC6174603

[B23] Pedrero E. J. Rodríguez M. T. Ruiz J. M. (2012). Adicción o abuso del teléfono móvil. Revisión de la literatura. Adicciones 24, 139–152. 10.20882/adicciones.107 22648317

[B24] Pedrero-Pérez E. J. Rojo-Mota G. Huertas-Hoyas E. (2022). Systematic review: Treatment modalities applied in smartphone addiction/abuse. Health Addict. 22, 122–131. 10.21134/haaj.v22i1.637

[B25] Petry N. M. O'Brien C. P. (2013). Internet gaming disorder and the DSM-5. Addiction 108, 1186–1187. 10.1111/add.12162 23668389

[B26] Shaw H. Ellis D. A. Geyer K. Davidson B. I. Ziegler F. V. Smith A. (2020). Quantifying smartphone “use”: Choice of measurement impacts relationships between “usage” and health. Technol. Mind Behav. 1, 1–15. 10.1037/tmb0000022

[B27] Shotton M. A. (1989). Computer addiction. A study of computer dependency. Taylor and Francis. London: Taylor & Francis Group.

[B28] Wikipedia (2022). Cigarette. Wikipedia. Available online at: https://en.wikipedia.org/wiki/Cigarette (accessed April 12, 2022).

